# Data-driven exploration of Na–Bi compounds: a first-principles and machine learning approach to topological thermoelectrics

**DOI:** 10.1039/d5ra05888k

**Published:** 2025-11-03

**Authors:** Souraya Goumri-Said, Houssam Eddine Hailouf, Khalid Reggab, Kingsley Onyebuchi Obodo, Mohammed Benali Kanoun

**Affiliations:** a College of Science and General Studies, Department of Physics, Alfaisal University P.O. Box 5092 Riyadh 11533 Saudi Arabia sosaid@alfaisal.edu; b Materials Science and Informatics Laboratory, Faculty of Science, University of Djelfa 17000 Djelfa Algeria; c School of Agriculture & Science, University of KwaZulu-Natal Pietermaritzburg Scottsville 3209 South Africa; d Center for Space Research, North-West University Private Bag X6001 Potchefstroom 2531 South Africa; e Department of Mathematics and Sciences, College of Sciences and Humanities, Prince Sultan University P.O. Box 66833 Riyadh 11586 Saudi Arabia

## Abstract

We conduct an in-depth investigation of the structural, electronic, vibrational, thermodynamic, and thermoelectric characteristics of Na–Bi-based compounds, specifically tetragonal NaBi, hexagonal NaBi_3_, and cubic Na_3_Bi, using advanced first-principles calculations in conjunction with machine learning (ML) models. We used density functional theory (DFT) with spin–orbit coupling (SOC) to figure out the electronic structure, phonon dispersions, and thermoelectric transport using Boltzmann transport theory. Our findings validate the Dirac semimetal nature of cubic Na_3_Bi and demonstrate varied topological and thermodynamic properties within the Na–Bi family. To speed up the prediction of the thermoelectric figure of merit (*ZT*) while enhancing interpretability to understand at the feature level, we trained supervised ML models [Random Forest (RF) and Neural Network (NN)] on thermoelectric results from DFT. It is possible to directly compute the figure of merit (*ZT*) from DFT-derived transport coefficients such as the Seebeck coefficient, electrical conductivity, and thermal conductivity. However, machine learning (ML) models serve as powerful surrogate predictors, enabling rapid screening of derivative compounds and quantitative assessment of feature importance through SHAP (SHapley Additive exPlanations) analysis. At low temperatures, RF models consistently outperformed NN models, but both performed well at high temperatures. SHAP analysis showed that the Seebeck coefficient has the biggest effect on *ZT* in all regimes. This integrated, physics-informed, and data-driven methodology demonstrates that machine learning can significantly augment first-principles approaches. It accelerates predictions, guides feature prioritisation, and enhances design capabilities. The developed workflow provides a generalizable and interpretable framework for the predictive modeling of advanced topological thermoelectric materials.

## Abstract 2

We investigate the structural, electronic, vibrational, thermodynamic, and thermoelectric properties of Na–Bi compounds (NaBi, NaBi_3_, Na_3_Bi) using advanced first-principles calculations combined with machine learning models.

## Graphical abstract

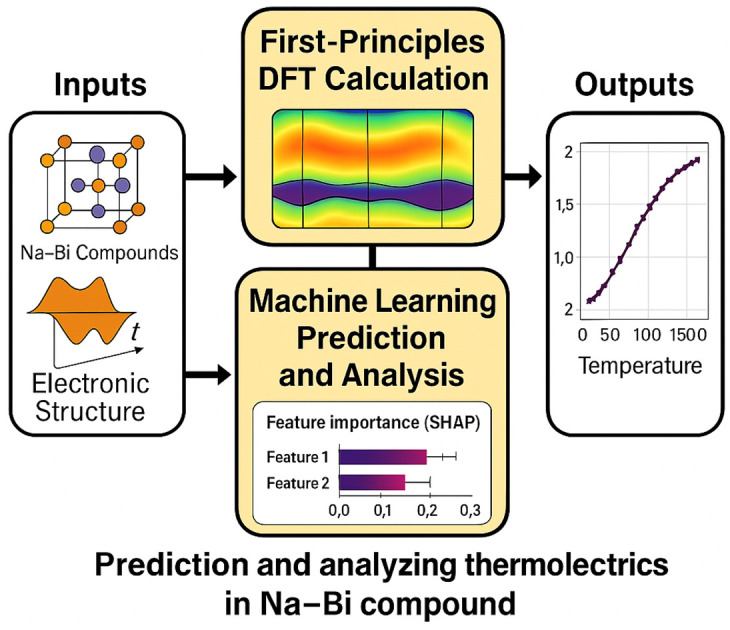

## 1. Introduction

Topological quantum materials, particularly insulators and semimetals, have attracted considerable attention for their unique band structures arising from inverted bulk bands and relativistic fermion states. Among these, sodium bismuthide compounds, including trisodium bismuthide (Na_3_Bi) and equiatomic NaBi, have emerged as prototypical systems for exploring such phenomena. Na_3_Bi was the first three-dimensional (3D) Dirac semimetal to be confirmed. It has symmetry-protected Dirac nodes along the *k*_*x*_ axis of the Brillouin zone, where bulk conduction and valence bands (mostly Na 3s and Bi 6p) are flipped because of strong spin–orbit coupling (SOC).^1,2^ When time-reversal or inversion symmetries are broken, these Dirac points can split into Weyl nodes. This is a classic example of Dirac semimetal behaviour.^3^ This leads to observable transport phenomena, including negative longitudinal magnetoresistance due to the chiral anomaly and weak anti-localization indicative of the π Berry phase of the Dirac fermions.^1^ Na_3_Bi also has a unique tunable optical response in the infrared, with a mid-infrared transparency window that can be changed by changing the carrier density. This shows that it could be useful for new optoelectronic applications.^4,5^ On the other hand, NaBi is a “topological metal” because it has a bulk band inversion and a nontrivial Z_2_ topology, even though it is metallic.^2^ Its SOC-driven inverted bands create Dirac-like surface states, which means it is in between topological insulators and metals. NaBi also has unique lattice dynamics, such as predicted ultralow and anisotropic lattice thermal conductivity and a closeness to superconductivity through electron–phonon coupling. This makes it a good platform for studying how topology, lattice vibrations, and superconductivity work together.^2^ Both Na_3_Bi and NaBi can change their topological phases when pressure is applied. Na_3_Bi transitions from a Dirac semimetal to a cubic phase at ∼0.8 GPa, maintaining band inversion akin to HgTe, and eventually becomes a trivial insulator at >118 GPa.^3^ Similarly, NaBi transforms into a cubic phase above ∼36 GPa, with anticipated modifications to its topological character.^3^ These transitions illustrate the critical link between crystal structure and band topology. Chemical alloying and dimensional reduction further enrich the phase diagram of Na–Bi compounds. In Na_3_Bi_1−*x*_Sb_*x*_ alloys, increasing Sb content reduces the effective SOC, driving a topological phase transition to a trivial insulator.^6^ Likewise, ultrathin Na_3_Bi films exhibit a thickness-dependent crossover from a 3D Dirac semimetal to a 2D quantum spin Hall (QSH) insulator state, with helical edge states emerging at sub-four-monolayer thicknesses.^4^ This tunability makes Na–Bi materials attractive candidates for low-power electronic and spintronic devices.

The goal of this study is to use a combination of first-principles density functional theory (DFT) and machine learning (ML) models to look at the structural, electronic, vibrational, thermodynamic, and thermoelectric properties of Na–Bi compounds. These include tetragonal NaBi, hexagonal NaBi_3_, and cubic Na_3_Bi. The goal is to find out if they could be used as topological thermoelectric materials and develop a suitable method to predict how effectively they will work as thermoelectric materials. In addition to first-principles thermoelectric modeling, this work integrates supervised machine learning (ML) to predict *ZT* from key transport descriptors (Seebeck coefficient, electrical and thermal conductivity). While *ZT* can be computed directly, this ML framework is introduced to (i) enable rapid prediction and screening of thermoelectric behavior once features are known, (ii) quantify the relative impact of each transport parameter on *ZT* using SHAP interpretability analysis, and (iii) demonstrate the feasibility of physics-informed surrogate modeling. This proof-of-concept provides a pathway for extending ML prediction to Na–Bi alloys, doped variants, or related compounds where DFT calculations are expensive or incomplete. The paper is organized as follows: Section 2 describes the computational methods employed for DFT, phonon, thermodynamic, and transport property calculations, along with the implementation of ML models. Section 3 presents and discusses the results, including structural optimization, electronic band structures, phonon dispersions, thermodynamic behavior, thermoelectric properties, and ML-based prediction and feature analysis. The key conclusions and future directions are summarized in Section 4.

## 2 Theoretical methodologies and computational details

### 2.1 Density functional theory

In this work, spin–orbit coupling (SOC)-inclusive density functional theory (DFT) calculations were carried out using the plane-wave-based Quantum ESPRESSO package.^7^ To precisely capture electron–ion interactions, we employed both ultrasoft^8^ and norm-conserving Vanderbilt pseudopotentials,^9^ utilizing fully relativistic large-core formulations.^10^ Our simulations were conducted on NaBi in its tetragonal, NaBi_3_ in its hexagonal, and Na_3_Bi in its cubic phases. The valence electron configurations for the elements under investigation were treated as follows: Na: 3s^1^, and Bi: 6s^2^6p^3^. For geometric relaxation, the exchange correlation GGA-PBE + SOC^11^ functional was employed. Structural optimization was carried out using the Broyden–Fletcher–Goldfarb–Shanno (BFGS)^12^ approach, employing a 12 × 12 × 8 Monkhorst–Pack grid.^13^ To ensure energy convergence, we imposed the condition that the major component of Hellmann–Feynman forces acting on a single atom must be less than 1 × 10^−10^ eV Å^−1^. Furthermore, to elucidate crystal structure and stability, we employed hybrid Heyd–Scuseria–Ernzerhof (HSE)^14^ computations, which incorporate SOC effects and an adaptively compressed exchange operator to enhance computational efficiency. Subsequently, we investigated the electronic and optical properties of the materials under study. Band structures incorporating SOC effects were computed using the HSE + SOC method, with Wannier functions^15^ interpolated using the Wannier Tools code.^16^

### 2.2 Thermodynamic properties

In order to determine the lattice dynamical stabilities for materials, the phonon dispersion (
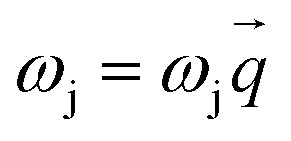
 for the association between 
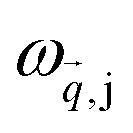
 and 
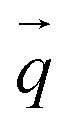
 for each j mode) is studied as the frequency dependance on the wave vector attained within linear response theory/density functional perturbation theory (DFPT) to obtain the response to periodic perturbations.^17^ The phonon density of states *g*(*ω*) gives the frequency distribution of normal modes and is calculated as:1
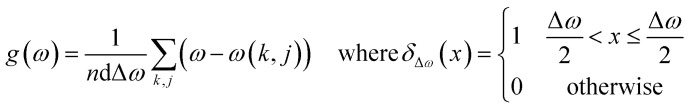


Thermodynamic favorability is determined by a relative free energy, so that the Helmholtz free energy is favored over Gibbs free energy for its universality of expressing reversible work at constant temperatures. The Helmholtz free energy for a nonmagnetic ideal crystal is expressed as:^18,19^

2 *A*(*V*,*T*) = *U*_0_(*V*) + *A*_el_(*V*,*T*) + *A*_phon_(*V*,*T*)

where *U*_0_(*V*) is the total energy, *A*_el_(*V*,*T*) is the electronic excitation contribution and *A*_phon_(*V*,*T*) is the nuclear vibrational motion or the phonon vibration contribution from phonon DOS *g*(*ω*) which is dependent the temperature. The vibrational partition function is given by:3
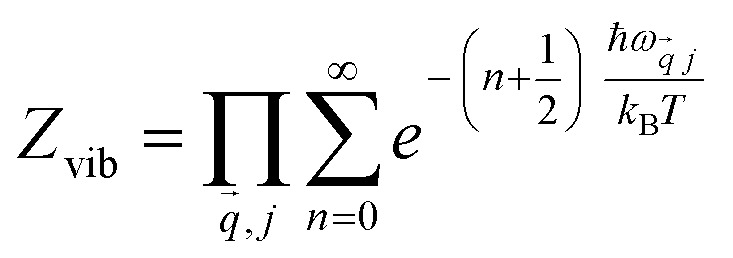


Therefore, the vibrational Helmholtz energy in the harmonic approximation is formulated as:4
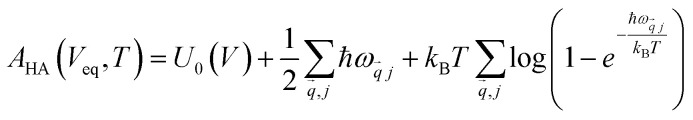


It relies on the temperature only by phonon contributions calculated for the equilibrium volume *V*_eq_. The amplitude of the vibrations is eased, thus validating the harmonic approximation for practically all situations at low temperatures. The isochoric heat capacity (*C*_*ν*_) for *N*_a_ atoms per unit cell is:5
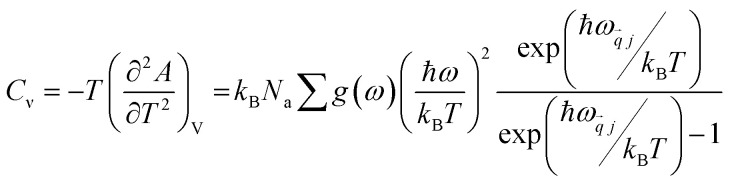


Yet, the truncation of the third term ignores the anharmonicity in the total energies, which causes some errors including, infinite values in thermal conductivity and phonon lifetimes as well as null quantities for the thermal expansion.^18^ Thus, considering temperature effects alongside vibrational degrees of freedom requires assuming a rigid harmonic approximation for the geometry, even as the crystal structure deviates from equilibrium (*i.e.* to some degree encompassing anharmonic contributions).^20^ This technique is referred to as the quasi-harmonic approximation (QHA) which presents the computation of phonons for multiple volumes 
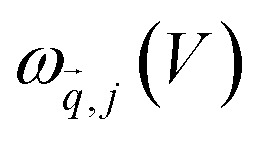
 so that the Helmholtz free energy turns into:6
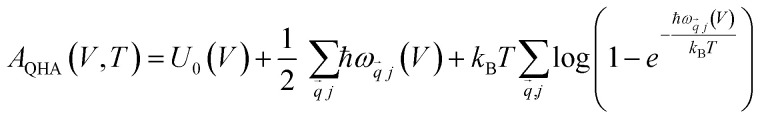
where the first term and second term are grouped as the cold potential energy *U*_cold_(*V*) at *T* = 0K and the second term is the thermal factor contributed by the phonons *A*_th_(*V*) which becomes negligible at extremely low *T*. Thus, the entropy (*S*) where the phonon frequency is volume dependent, with the neglect of the temperature dependent intrinsic phonon interactions, is written as:^21^7
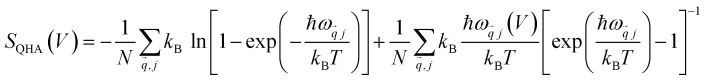


The thermodynamical modeling procedure which exhibit a comprehensive DOS for the vibrational modes is achieved through the Debye model which is simplified to:^19,20^

8 *g*(*ω*) = *C*·*ω*^2^·Θ(*ω* − *ω*_D_)

where Θ is the Heaviside step function, and C is a constant for the 
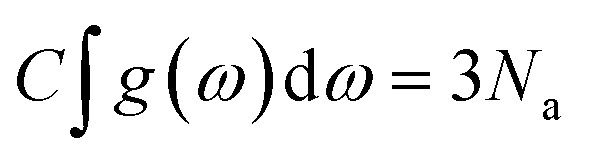
 is expressed as *C* = 9*N*_a_/*ω*^3^_D_. The term *ω*_D_ is the Debye frequency, wherein the phonon modes are populated below it. The determination of the phonon Helmholtz free energy and the isochoric heat capacity in the Debye model are represented as:9
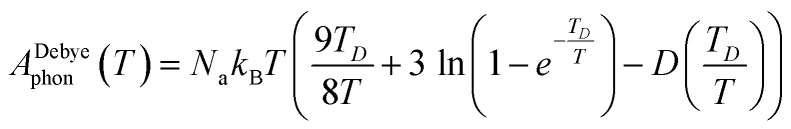
10
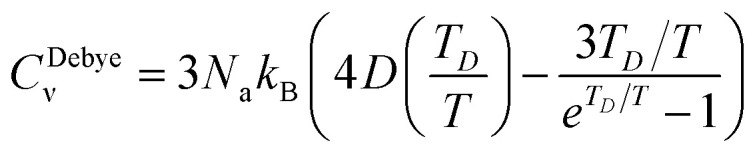
where 
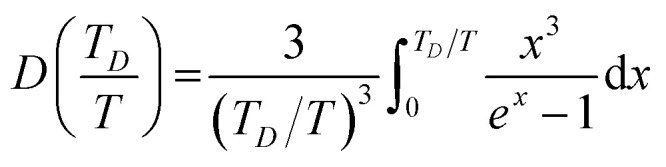
 is the Debye integral and
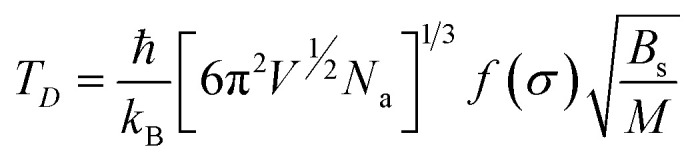
 is the Debye temperature which is the temperature where every mode under the highest frequency mode *ω*_D_ is excited (maximum phonon frequency).^22^

### 2.3 Thermoelectric properties: boltzman transport theory

The thermoelectric characteristics are evaluated using the semi-classical Boltzmann transport theory, implemented *via* the BoltzTrap software in combination with first-principles calculations and the relaxation time approximation.^23^ This approach models the statistical behavior of charge carriers in systems slightly deviating from thermal equilibrium. The foundation of the Boltzmann transport equation lies in the assumption that particles (such as electrons) undergo random motion within the material. To overcome the difficulty in estimating the relaxation time, the deformation potential theory is used in a theoretical context.

The electronic transport is defined through the electrical and heat currents *J*_e_ and *J*_Q_ as:

11 *J*_e_ = *σE* − *σS*∇*T*

12 *J*_Q_=*TσSE* − *κ*_0_∇*T*

where *σ* is the electrical conductivity, *S* is the Seebeck coefficient, *κ* is the thermal conductivity, *E* is the electric field, and ∇*T* is the temperature gradient. By expressing a simplified transport distribution Ξ, the transport properties are easily computed through:13
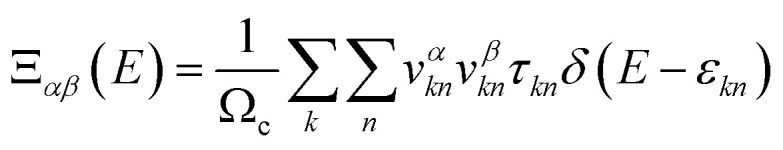


The *σ* and *S* are computed obeying equations which are termed as a function of chemical potential (*μ*) and temperature (*T*).^24^14
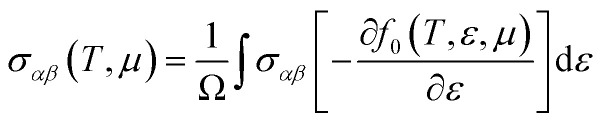
15
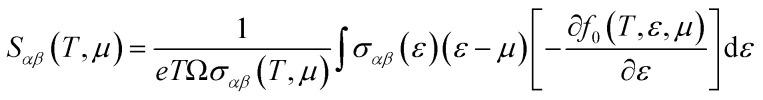
16
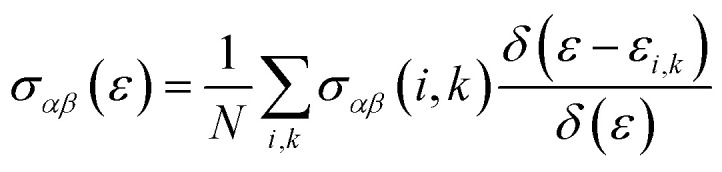
17
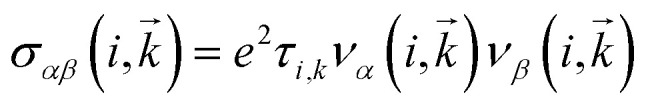
Here, *α* and *β* denote tensor components, *e* represents the elementary charge, and *f*_0_ is the Fermi-Dirac distribution function. The symbol Ω refers to the volume of the unit cell, *μ* is the chemical potential, and *τ* is the relaxation time, assumed constant at 10^−14^ s. The group velocity components, indicated as 
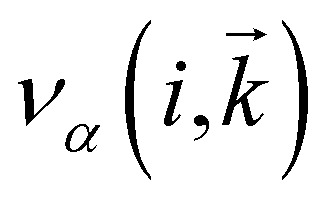
, are obtained from the electronic band structure. When τ is considered constant and independent of the wave vector 
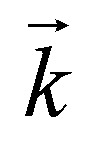
 and carrier energy, it cancels out in the ratio between the two integrals used in the Seebeck coefficient calculation.^25^ The use of a constant relaxation time (*τ* = 10^−14^ s) is a standard and practical approximation in Boltzmann transport modeling, particularly for high-throughput screening, as it enables a focus on the intrinsic electronic-structure-driven trends that govern thermoelectric behavior.^26^ This assumption is most appropriate for scattering mechanisms with weak energy dependence; such as neutral impurity or alloy scattering; and serves as a reasonable first-order approximation at elevated temperatures where acoustic phonon scattering dominates and its energy dependence becomes less pronounced. While this simplification omits the full complexity of energy-dependent scattering from acoustic and optical phonons or ionized centers,^27,28^ it remains sufficient for the comparative analysis pursued here. As discussed by Singh and co-workers,^29^ BoltzTraP-based calculations under constant *τ* are most reliable for identifying qualitative and relative trends in transport properties rather than absolute *ZT* magnitudes, which may deviate near the optimized chemical potential where multiple scattering channels compete. Therefore, the *ZT* values reported in this work represent indicative trends, not experimentally calibrated maxima, and the large Seebeck coefficients observed for Na_3_Bi arise directly from its Dirac-like band dispersion and low density of states near the Fermi level.

### 2.4 Machine learning models

Random Forest is a non-parametric ensemble learning method^30,31^ composed of multiple decision trees, each trained on a bootstrap sample of the data with randomly selected subsets of features. The regression prediction is an average over all trees:^32,33^18
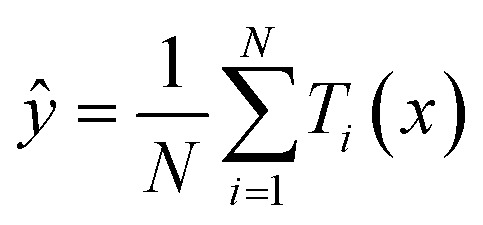
where, *ŷ* predicted output (*e.g.*, *ZT*), *N*: number of trees in the forest, *T*_i_(*x*): output of the i-th decision tree for input *x*.

Neural Networks,^34,35^ by contrast, consist of layers of interconnected nodes that transform the input through weighted sums and nonlinear activation functions. The basic structure used in this study is:

19 ŷ = *f*(*W*_2_·ReLU(*W*_1_*x* + *b*_1_) + *b*_2_)

where, *x*: input feature vector (*e.g.*, *S*, *σ*, *κ*), *W*_1_, *W*_2_: weight matrices.,*b*_1_, *b*_2_: bias vectors, ReLU(*z*) = max(0,*z*): activation function, *f*: output activation (identity for regression), *ŷ*: predicted *ZT*.

Neural networks can capture complex, highly nonlinear relationships, but require careful regularization and sufficient data to avoid overfitting.

## 3. Results and discussion

### 3.1 Crystal structure and optimization

**Fig. 1:**
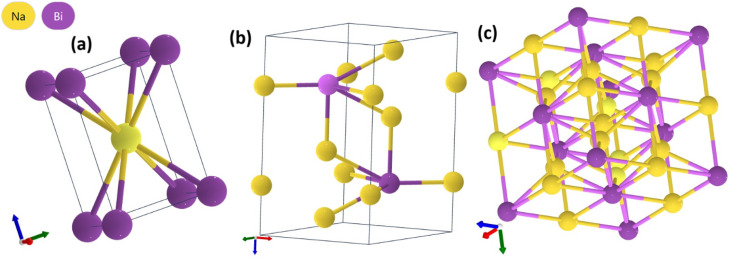
Optimized crystals structures of (a) NaBi tetragonal, (b) NaBi_3_ hexagonal and (c) cubic Na_3_Bi using GGA-PBE + SOC.

Fig. 1 shows the optimized crystal structures of (a) tetragonal NaBi, (b) hexagonal NaBi_3_, and (c) cubic Na_3_Bi, obtained through first-principles calculations using density functional theory (DFT) with spin–orbit coupling (SOC). Structural relaxations were performed using the Broyden–Fletcher–Goldfarb–Shanno (BFGS) algorithm^36^ until the total energy converged below 10^−8^ Ry and Hellmann–Feynman forces on each atom dropped below 10^−4^ Ry Bohr^−1^.

To make sure that the forces, stress, and electronic properties converged correctly, we used a plane-wave energy cutoff of 80 Ry and a Monkhorst–Pack grid of 12 × 12 × 8.^37^Table 1 shows the optimised lattice constants and the thermodynamic properties that go with them. The relaxed lattice parameters for tetragonal NaBi were *a* = 3.4052 Å, *c* = 4.8671 Å, and the *c*/*a* ratio was 1.4293. This corresponds to a unit cell volume of 56.44 Å^3^. The predicted formation energy is −0.381 eV/atom, which means that the system is moderately thermodynamically stable. Hexagonal NaBi_3_, on the other hand, has *a* = 6.9326 Å, *c* = 5.7899 Å, and a lower *c*/*a* ratio of 0.8351. It also has a much larger volume of 240.98 Å^3^ and a more negative formation energy of −0.498 eV per atom, indicating higher thermodynamic stability. The cubic Na_3_Bi structure has a lattice constant of *a* = 7.6478 Å, a volume of 447.31 Å^3^, and a formation energy of −0.482 eV per atom. This is in line with previous experimental and theoretical studies that demonstrated it was a very stable 3D Dirac semimetal.^38,39^ We employed the standard method for determining out the formation energies:20
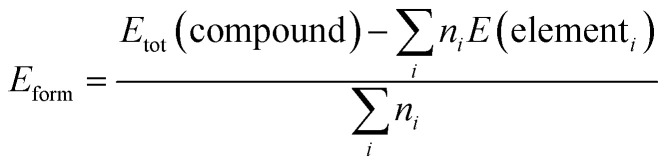
where *E*_tot_ is the total energy of the compound, *n*_*i*_ is the number of atoms of element *i*, and *E*(element_*i*_) is the energy of each elemental reference in its standard state. All three phases have negative formation energies, which means that the compounds are thermodynamically stable against breaking down into their constituent elements. This is in line with the rules set out in materials discovery frameworks like the Materials Project^40^ and AFLOW.^41^ The rising formation energy from NaBi to NaBi_3_ suggests that adding more Bi atoms could make the structure more stable by making the Na–Bi bonds stronger and spreading the charge more evenly. These results show that the Na–Bi family has a wide range of structures and is stable when it comes to thermodynamics. This gives us a reason to look into their electronic, topological, and optoelectronic properties more closely.

**Table 1:** Optimized lattice parameters (*a*, *c*, *c*/*a*), unit cell volumes, and formation energies of NaBi (tetragonal), NaBi_3_ (hexagonal), and Na_3_Bi (cubic) from DFT-GGA + SOC calculations. Experimental lattice constants (where available) are included for comparison^a^

Compound & structure	Source	*a* (Å)	*c* (Å)	*c*/*a*	Volume (Å^3^)	Formation energy (eV per atom)
NaBi – tetragonal (*P*4/*mmm*)	This work (DFT)	3.4052	4.8671	1.4293	56.4359	−0.381
	Exp^44^	3.46	4.80	1.39	57.5	−0.329
	DFT^44^	3.42	4.89	1.43	57.6	−0.366
NaBi_3_ – hexagonal (*P*6_3_/*mmc*)	This work (DFT)	6.9326	5.7899	0.8351	240.9801	−0.498
	Exp^38^	5.448	9.655	1.77	248.2	−0.495
	DFT^38^	5.458	9.704	1.78	251.0	−0.367
Na_3_Bi – hexagonal (*P*6_3_/*mmc*)	DFT^43^	5.448	9.655	1.77	248.2	—
	DFT^45^	5.37	9.64	1.80	240.7	—
Na_3_Bi – cubic (*Fm*3̄*m*)	This work (DFT)	7.6478	—	—	447.3109	−0.482

a Note: Na_3_Bi is typically reported in hexagonal structure (*P6*_3_/*mmc*) in literature. This study focuses on the cubic polymorph (*Fm*3̄*m*).

To validate the reliability of our DFT-GGA + SOC approach, we compared the optimized lattice parameters with available experimental values, as shown in Table 1. The deviations are within ±1%, which shows that the structural models used for later electronic and thermoelectric analyses are correct. These minor discrepancies are characteristic of GGA-based functionals and align with prior experimental and theoretical research on Na–Bi systems.^42–48^ This comparison strengthens the accuracy of our theoretical framework in capturing the essential structural characteristics of Na–Bi compounds.

### 3.2 Electronic properties

**Fig. 2:**
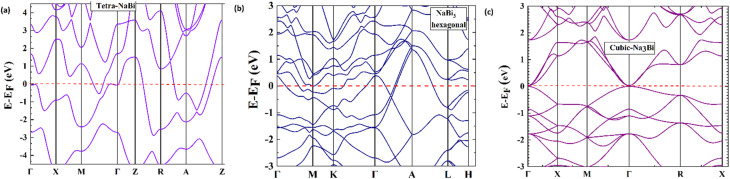
The electronic band structure of (a) NaBi tetragonal, (b) NaBi_3_ hexagonal and (c) cubic Na_3_Bi calculated *via* HSE + SOC. The horizontal dashed line indicates the Fermi level, which is set at the zero level.

Fig. 2 shows the electronic band structures of three Na–Bi-based compounds: tetragonal NaBi, hexagonal NaBi_3_, and cubic Na_3_Bi. These were calculated using the HSE06 hybrid functional with spin–orbit coupling (SOC). For tetragonal NaBi (Fig. 2a), the Fermi level is in a band that is only partially filled, which means that the material exhibits metallic or semimetallic behavior. The fact that there is band inversion near the *Γ*-point because Bi has a strong SOC means that the material may have nontrivial topological states, which makes it a possible topological semimetal. The hexagonal NaBi_3_ phase (Fig. 2b) has a band structure that is both dense and highly dispersive, with many crossings at the Fermi level. This suggests that it is a metal. The bands near high-symmetry points (*Γ*, *A*, *L*) are very complicated, which implies that the Fermi surface is quite rich. There is no clear band gap, but it is still possible that the material has nodal-line or Dirac semimetal character, suggesting the need for further topological surface state analysis.

In contrast, cubic Na_3_Bi (Fig. 2c) shows a well-defined Dirac crossing along the *Γ*–*Z* direction, slightly above the Fermi level. This feature is symmetry-protected and robust under SOC, confirming Na_3_Bi's status as a prototypical three-dimensional Dirac semimetal, as previously reported by Shao *et al.*^49^ and confirmed experimentally by Liu *et al.*^1^ Na_3_Bi is frequently compared to Cd_3_As_2_, another 3D Dirac semimetal, which shows similar linear dispersion and topological protection when inversion and time-reversal symmetry are present.^50,51^ The effect of SOC is significant in all three phases, causing band inversions and topological features. These findings underscore the Na–Bi material family as a promising foundation for adjusting topological phases through crystal symmetry and indicate the possibility of achieving innovative quantum phenomena through structural engineering.

**Fig. 3:**
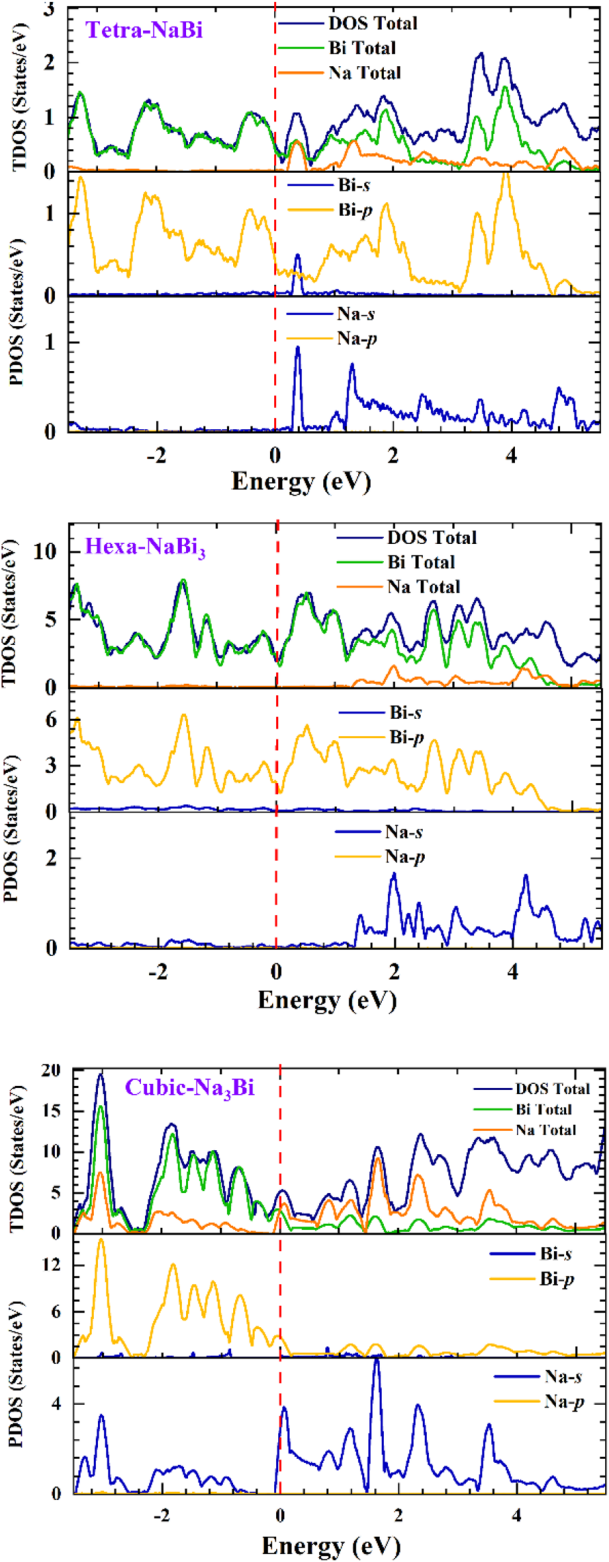
The electronic TDOS and PDOS for (a) NaBi tetragonal, (b) NaBi_3_ hexagonal and (c) cubic Na_3_Bi calculated *via* HSE + SOC. The horizontal dashed line indicates the Fermi level, which is set at the zero level.

Fig. 3 shows the total and projected density of states (TDOS and PDOS) for tetragonal NaBi, hexagonal NaBi_3_, and cubic Na_3_Bi. This gives us information about their electronic structure and how their orbitals contribute. In tetragonal NaBi (Fig. 3a), the total density of states (DOS) exhibits a finite value at the Fermi level (*E*_F_ = 0 eV), thereby affirming its metallic character, which is consistent with the band structure depicted in Fig. 2a. The PDOS shows that the states close to the Fermi level mostly come from Bi-6p orbitals, and Na only adds a little bit. The prevalence of Bi-p states aligns with the potential for SOC-induced band inversion, a critical hallmark of topological semimetal characteristics.

In the case of hexagonal NaBi_3_ (Fig. 3b), the TDOS is significantly higher than in the tetragonal phase, and the sharp Bi-p peaks below the Fermi level highlight strong hybridization and orbital density. Like the tetragonal phase, the Na states stay close to the Fermi level, while the Bi-p orbitals are the main contributors close to it. This strengthens the idea that the material is a complex metal and suggests at the possibility of Dirac or nodal-line semimetallic properties. The DOS profile for cubic Na_3_Bi (Fig. 3c) confirms about its Dirac semimetal behaviour. There is a strong peak of Bi-p orbitals close to the Fermi level. This peak is very important in the formation of the symmetry-protected Dirac nodes, demonstrated by the band crossing in Fig. 2c. Na-s orbitals contribute mostly in the conduction band region and are not involved in low-energy excitations. The prevalent presence of Bi-derived p-states in all three structures highlights Bi's essential function in affecting the electronic topology of these compounds. These results align with prior research on Na_3_Bi and comparable topological materials, wherein heavy-element p-orbital states dominate at the band edges, facilitating SOC-induced nontrivial phases.^49–51^

### 3.3 Phonon, vibrational study and thermodynamics

**Fig. 4:**
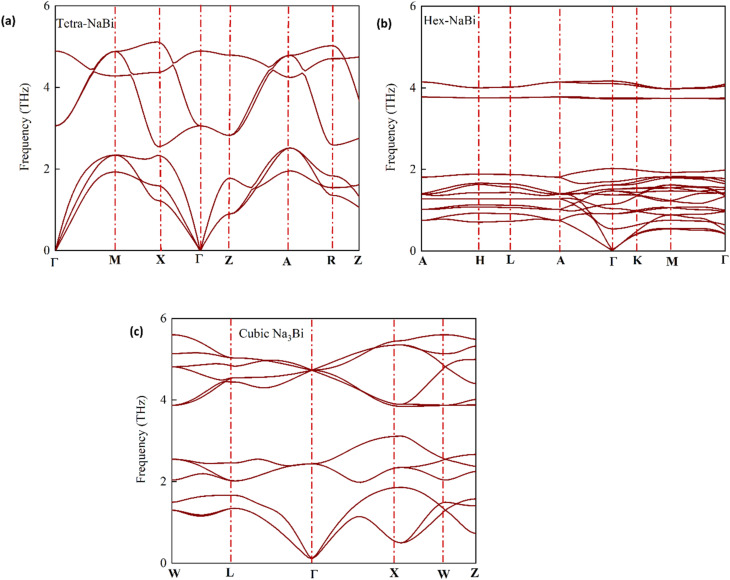
Phonon dispersion curves of (a) tetragonal NaBi, (b) hexagonal NaBi_3_, and (c) cubic Na_3_Bi phases.

Fig. 4 presents the calculated phonon dispersion curves for (a) tetragonal NaBi, (b) hexagonal NaBi_3_, and (c) cubic Na_3_Bi, obtained using density functional perturbation theory (DFPT). The absence of imaginary phonon frequencies in the Brillouin zones for all three structures confirms their dynamical stability at zero temperature. For tetragonal NaBi (Fig. 4a), the phonon branches cover a frequency range of about 5.5 THz, with clear differences between the acoustic and optical modes. The high optical phonon frequencies, which are above 4 THz, suggest that the bonds are strong, especially between Bi and Na atoms. For hexagonal NaBi_3_ (Fig. 4b), the phonon spectrum shows a denser distribution of modes below 4.5 THz, with flat bands that stand out, especially in the optical branches. These flat modes suggest localised vibrational states, possibly resulting from anisotropic or layered bonding environments, which align with the quasi-1D chain-like structural motif depicted in Fig. 1b. The phonon spectrum of cubic Na_3_Bi (Fig. 4c) is typical of a highly symmetric rocksalt-type lattice, and the phonon branches go up to almost 5.5 THz. The degeneracies at high-symmetry points like *Γ* and *X* show that the crystal has cubic symmetry. The clear separation of the acoustic and optical branches and no evidence of phonon softening are strong indicators of mechanical and vibrational stability. These phonon properties back up the structural findings and prove that the Na–Bi phases being studied are thermodynamically and dynamically stable. Furthermore, the observed dispersion characteristics, such as flat optical bands and symmetry-induced degeneracies, can directly affect thermal transport and electron–phonon coupling, which are essential for describing superconductivity, thermoelectric performance, or phonon-limited mobility in these topologically significant systems. Comparable behaviour has been observed in other Bi-containing compounds, wherein heavy elements and significant spin–orbit coupling affect both lattice dynamics and electronic structure.^52–54^

**Fig. 5:**
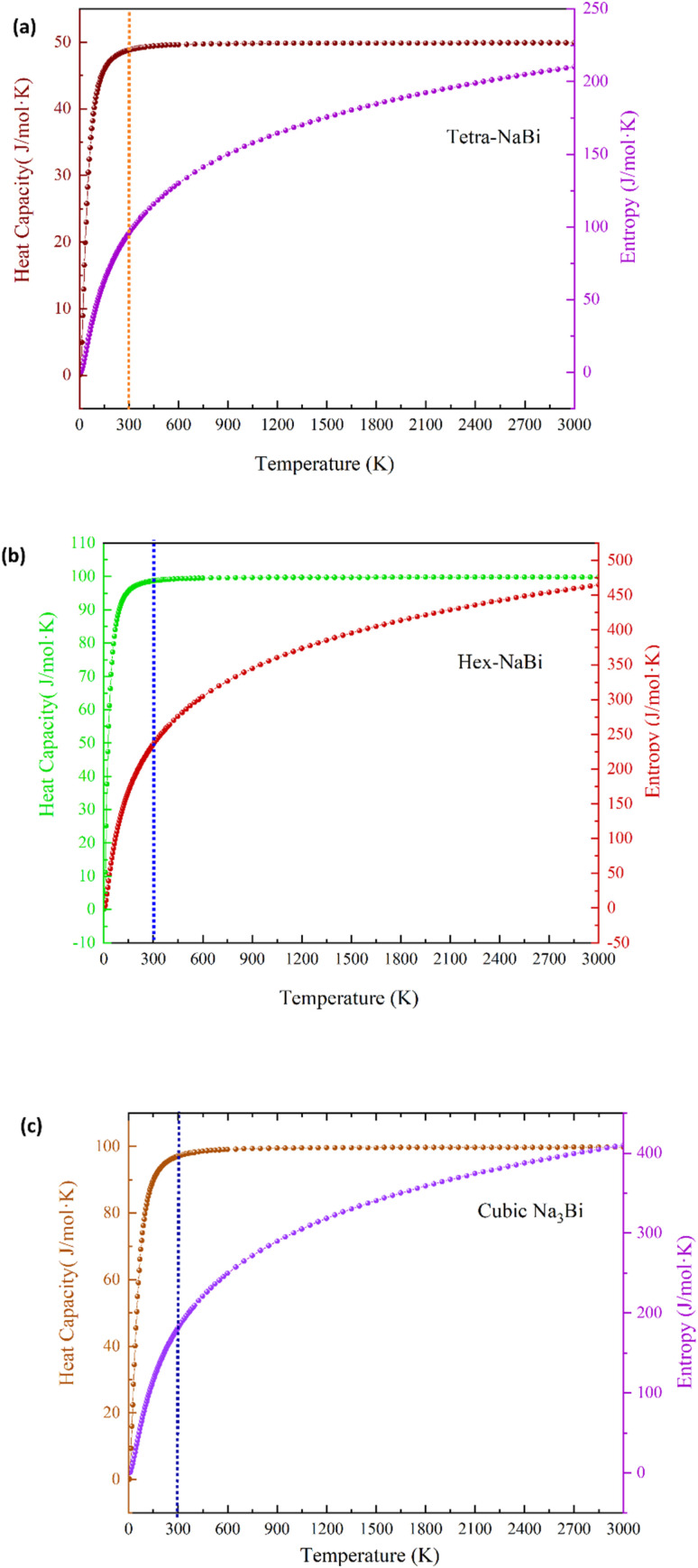
The temperature-dependent thermodynamic properties of (a) tetragonal NaBi, (b) hexagonal NaBi_3_, and (c) cubic Na_3_Bi were found using phonon calculations. The graphs show how heat capacity at constant volume (*C*_*ν*_) and entropy (*S*) change with temperature up to 3000 K. All compounds show the expected saturation of *C*_*ν*_ at high temperatures, which is in line with the Dulong–Petit limit. The entropy rises steadily as the temperature rises. NaBi_3_ has the highest values because it has more complex atoms.

**Fig. 6:**
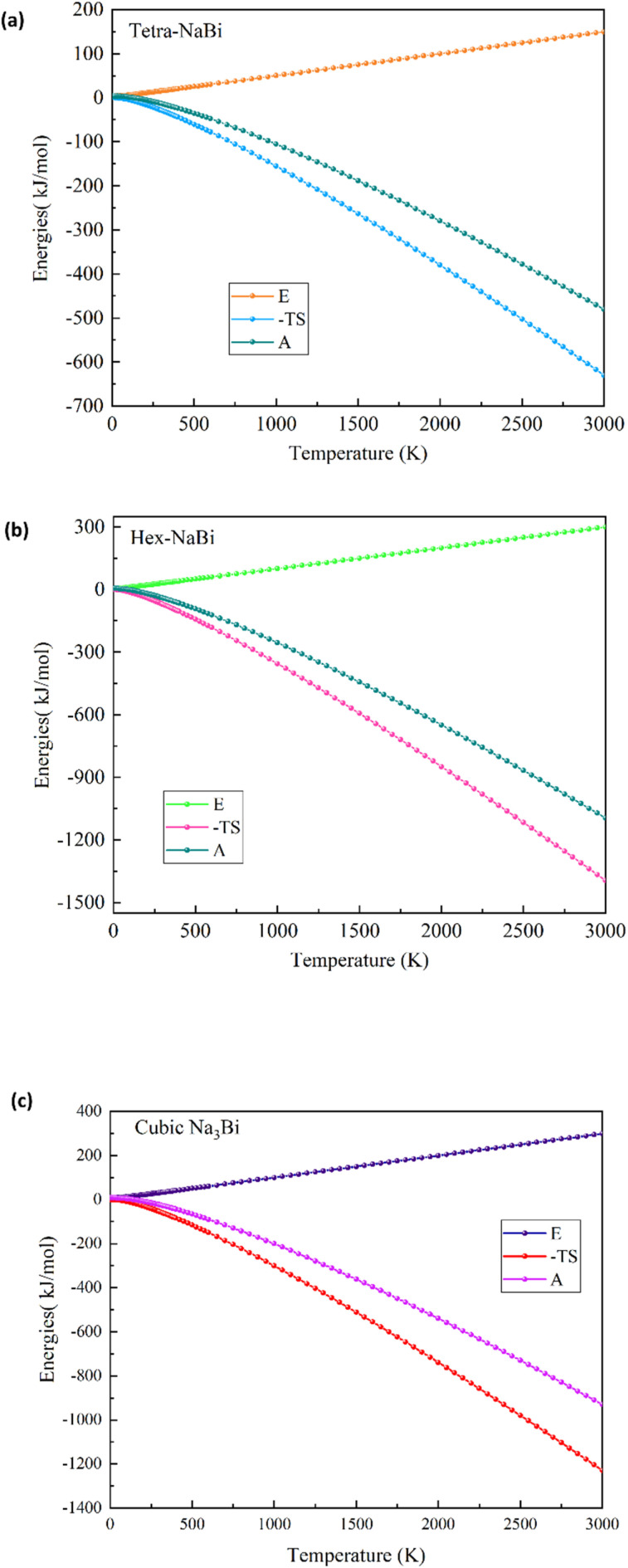
Helmholtz free energy (*A*), internal energy (*E*), and entropic contribution (–TS) *versus* temperature for (a) tetragonal NaBi, (b) hexagonal NaBi_3_, and (c) cubic Na_3_Bi. The free energy decreases with increasing temperature in all cases, driven primarily by the –TS term.

We used phonon-based quasiharmonic calculations to look at the thermodynamic stability and temperature-dependent behaviour of tetragonal NaBi, hexagonal NaBi_3_, and cubic Na_3_Bi. The results are shown in Fig. 5 and 6. Fig. 5 displays the computed heat capacity at constant volume (C_*ν*_) and entropy (*S*) up to 3000 K. All three structures show *C*_*ν*_ rising and then levelling off, which is what the Debye model suggests ought to occur. This means that all of the phonon modes are filled at higher temperatures. Tetragonal NaBi reaches a saturation *C*_*ν*_ of about 52 J mol^−1^ K^−1^, hexagonal NaBi_3_ goes over 100 J mol^−1^ K^−1^, and cubic Na_3_Bi stays stable at about 100 J mol^−1^ K^−1^. These trends show that the atoms in the series are getting increasingly complicated and have more ways to vibrate. Entropy increases monotonically with temperature, with hexagonal NaBi_3_ having the highest entropy (∼470 J mol^−1^ K^−1^ at 3000 K) because its unit cell is bigger and its phonon spectrum is richer.

Fig. 6 shows the Helmholtz free energy (*A* = *E* − TS), the internal energy (*E*), and the entropic contribution (−TS). With increasing temperature, the internal energy of all compounds increases while the Helmholtz free energy (*A*) decreases, highlighting the stabilizing role of entropy. NaBi_3_ has the most negative Helmholtz free energy at high temperatures, which supports its superior thermodynamic stability and is consistent with its lowest DFT-calculated formation energy (−0.498 eV per atom). The −TS term is very large in all three compounds, which shows that lattice entropy plays a big role in how they behave thermally. These trends correspond effectively with observations in structurally and chemically similar systems. Bi_2_Te_3_ and Sb_2_Te_3_, which are well-known thermoelectric materials, have similar *C*_*ν*_ saturation behaviour (about 125–130 J mol^−1^ K^−1^) and high entropy because they have heavy atoms and strong anharmonicity.^54,55^ PbTe, another standard thermoelectric, has similar levels of entropy (∼400–450 J mol^−1^ K^−1^) because it has soft optical phonons and polar bonding.^56^ Furthermore, NaSnBi, a topological compound structurally related to NaBi, exhibits thermal behavior governed by strong SOC and vibrational anisotropy.^57,58^ Similar phonon stability and free energy trends have also been reported for Na_3_Bi and Cd_3_As_2_, both known topological Dirac semimetals, reinforcing the predictive accuracy of the current phonon-based thermodynamic analysis.^59,60^

### 3.4 Thermoelectric properties

**Fig. 7:**
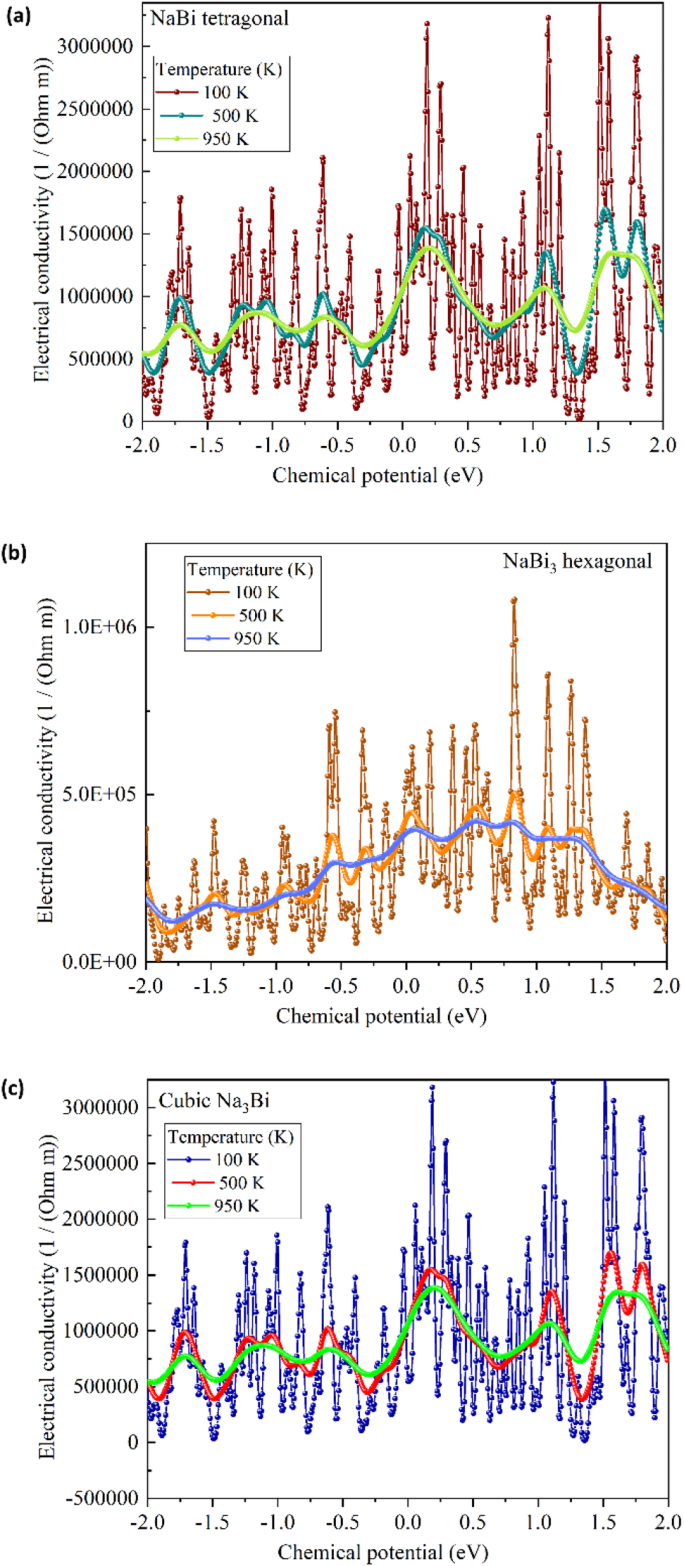
Calculated electrical conductivity (σ/τ) as a function of chemical potential for (a) tetragonal NaBi, (b) hexagonal NaBi_3_, and (c) cubic Na_3_Bi at 100 K, 500 K, and 950 K.

**Fig. 8:**
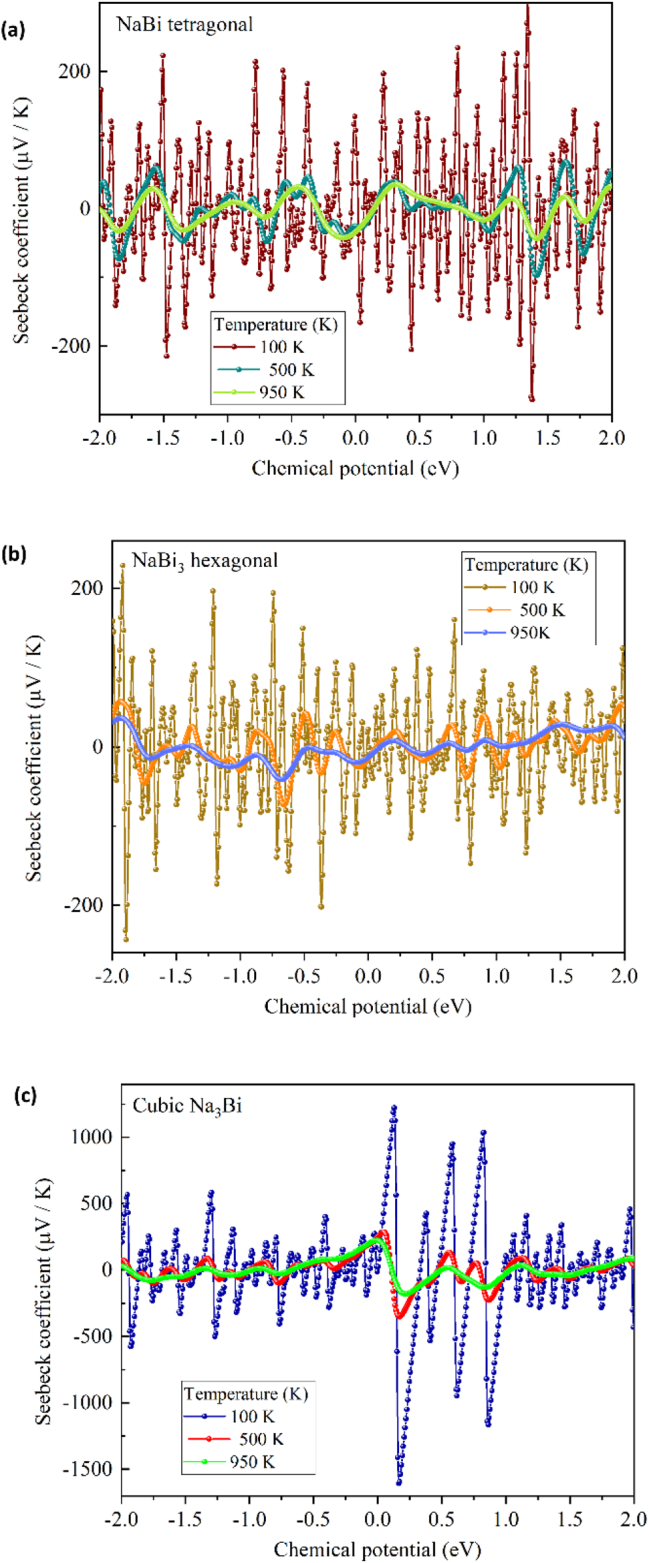
Seebeck coefficient (*S*) *versus* chemical potential for (a) tetragonal NaBi, (b) hexagonal NaBi_3_, and (c) cubic Na_3_Bi at 100 K, 500 K, and 950 K. Cubic Na_3_Bi shows highly enhanced and tunable Seebeck response with values exceeding ±1000 μV K^−1^ near the Dirac point, indicative of its topological semimetal nature.

**Fig. 9:**
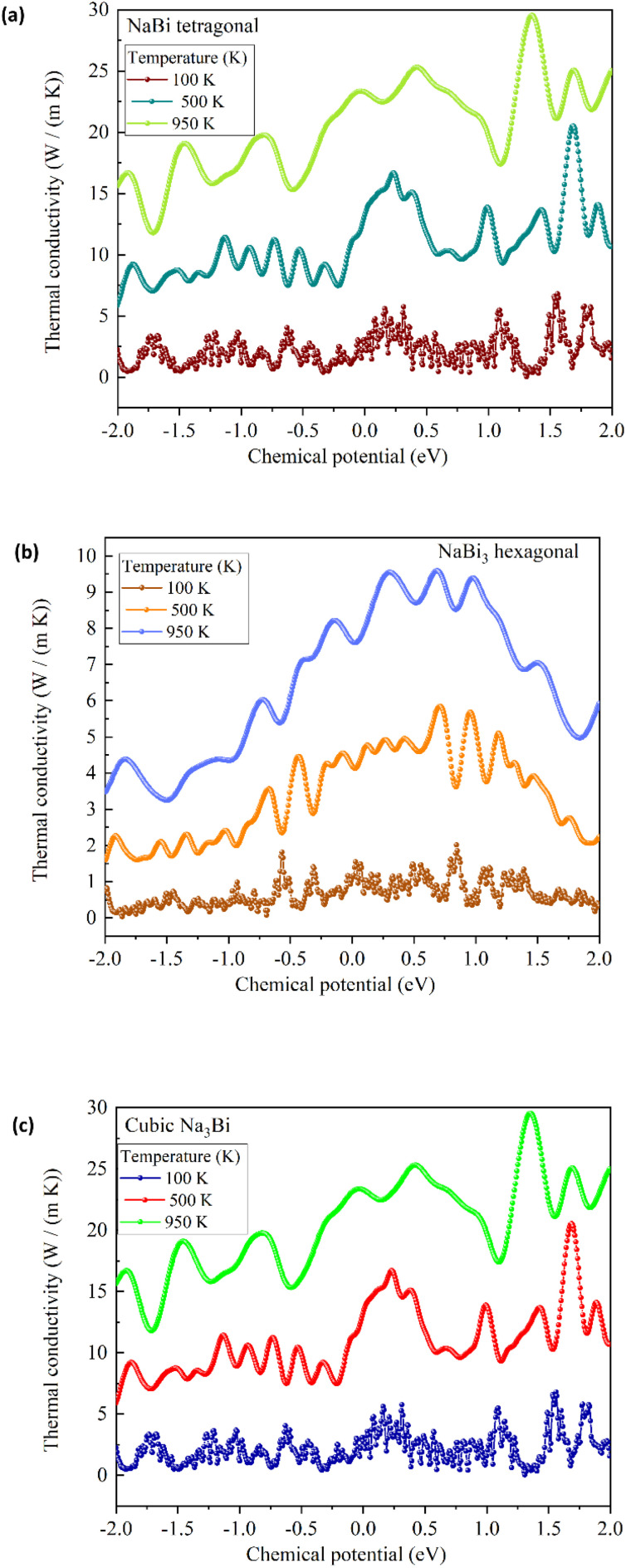
Electronic thermal conductivity (*κ*_e_) as a function of chemical potential for (a) tetragonal NaBi, (b) hexagonal NaBi_3_, and (c) cubic Na_3_Bi. Na_3_Bi and NaBi exhibit higher *κ*_e_ at high doping levels, while NaBi_3_ remains low across the range, advantageous for thermoelectric performance.

We used Boltzmann transport theory under the constant relaxation time approximation to look at the electronic transport coefficients of NaBi-based compounds in order to see the way that they performed as thermoelectric materials. Fig. 7, 8, and 9 respectively present the calculated electrical conductivity, Seebeck coefficient, and electronic thermal conductivity as functions of chemical potential at three representative temperatures: 100 K, 500 K, and 950 K. Fig. 7 shows the electrical conductivity (*σ*) behavior for tetragonal NaBi (a), hexagonal NaBi_3_ (b), and cubic Na_3_Bi (c). All three compounds show metallic properties, with *σ* values as high as 3 × 10^5^ (Ω m)^−1^ near the Fermi level, especially at low temperatures (100 K). Tetragonal NaBi and cubic Na_3_Bi exhibit elevated *σ* across a wide spectrum of chemical potential, which is ascribed to their more compact band structures in proximity to the Fermi level. As the temperature rises, thermal smearing dampens the amplitude of conductivity modulations. This is especially clear in hexagonal NaBi_3_, which has a relatively flat *σ* profile, which means that its electronic structure is less dispersive. Fig. 8 shows the Seebeck coefficient (*S*), which is an important number for thermoelectric applications. The Seebeck coefficients of tetragonal and hexagonal NaBi phases are moderate (∼±200 μV K^−1^), but cubic Na_3_Bi has very high and very tunable values, reaching up to ±1200 μV K^−1^ near the Fermi level at 100 K as its band crossings are linear and its dispersion is Dirac-like. These values are typical for topological semimetals, where bipolar conduction can greatly increase *S* in confined energy windows.^61^ The sign change of *S* around *μ* = 0 is crucial because it shows ambipolar behaviour, which means that both p-type and n-type conduction may occur depending on the doping. Among the three, Na_3_Bi demonstrates the most promising Seebeck response, especially under electron doping conditions. Fig. 9 depicts the electronic component of thermal conductivity (*κ*_e_), which increases with temperature and carrier concentration. Tetragonal NaBi and cubic Na_3_Bi exhibit *κ*_e_ values up to 25 W m^−1^ K^−1^ at 950 K near high-energy doping regimes, whereas hexagonal NaBi_3_ retains significantly lower values (<10 W m^−1^ K^−1^), which may be advantageous in reducing total thermal losses and enhancing *ZT* (thermoelectric figure of merit) when lattice contributions are also minimized. When comparing the three structures, cubic Na_3_Bi clearly exhibits superior thermoelectric characteristics, with high Seebeck coefficient, moderate to high electrical conductivity, and manageable electronic thermal conductivity. These findings, combined with its previously discussed topologically nontrivial electronic structure and dynamical stability, make it a strong candidate for topological thermoelectric applications. Hexagonal NaBi_3_, while less conductive, benefits from lower *κ*_e_, which can improve thermoelectric performance if lattice thermal conductivity is minimized. Tetragonal NaBi appears to lie between the two, with balanced but less extreme transport characteristics. These findings correspond with studies on topological materials like Bi_2_Te_3_, SnSe, ZrTe_5_, and Cd_3_As_2_, where mechanisms such as band convergence, Dirac dispersion, or band inversion result in enhanced Seebeck responses and optimised *ZT* values.^61–64^ Na_3_Bi has been recognised as a 3D Dirac semimetal exhibiting adjustable thermoelectric properties through alloying or strain engineering.^38,65^

To evaluate the thermoelectric efficiency of the investigated Na–Bi compounds, we calculated the dimensionless figure of merit 
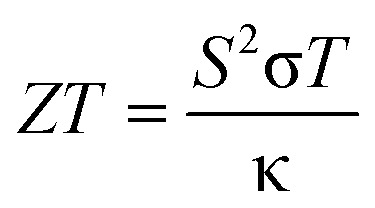
 at three representative temperatures: 100 K, 500 K, and 950 K. This was done using the extracted values of the *S*, *σ* and *κ*_e_ from our Boltzmann transport calculations. We assumed that all materials had a constant lattice thermal conductivity of 1.5 W mK^−1^, which is in line with what has been reported for Bi-based compounds. The resulting trends show that the three phases have very different thermoelectric behaviour. Among the investigated structures, cubic Na_3_Bi demonstrates the most promising thermoelectric performance at low and intermediate temperatures. It achieves a peak *ZT* of 0.53 at 500 K, driven by a combination of exceptionally high Seebeck coefficients (up to 650 μV K^−1^ at 100 K) and moderate electrical conductivity. However, its thermoelectric efficiency decreases to *ZT* ≈ 0.11 at 950 K due to a significant rise in electronic thermal conductivity, a known limitation of topological semimetals with metallic character. Tetragonal NaBi, on the other hand, exhibits more balanced behavior across the temperature range, reachinga maximum *ZT* of 0.29 at 500 K, which decreases to 0.13 at 950 K as *κ*_e_ increases and *S* saturates. Its relatively moderate Seebeck coefficient and stable *σ* allow it to maintain a reasonable *ZT* across the studied range. Hexagonal NaBi_3_ has the lowest *σ* and *S* values overall, but it has a lower *κ*_e_, which means that its *ZT* slowly but steadily improves with temperature, going from 0.03 at 100 K to 0.21 at 950 K. This means that although its electrical transport isn't as efficient, its lower thermal losses could make it useful in thermoelectric applications if more *κ*_l_ reduction methods (like alloying or nanostructuring) are used. These results show that cubic Na_3_Bi is the most suitable thermoelectric candidate below 600 K given that it has a Dirac-like band structure and a high Seebeck response. However, tetragonal NaBi and hexagonal NaBi_3_ may become more competitive for use at high temperatures because they have lower *κ*_e_ and a more stable thermoelectric response. These results are in line with what has been seen in other topological thermoelectric materials, like ZrTe_5_, Cd_3_As_2_, and engineered Na_3_Bi alloys, which show higher *ZT* through band structure tuning and phonon engineering. In general, the results show that the Na–Bi family can be tuned thermoelectrically and that there are many ways to improve their performance through electronic or phononic design.

### 3.5 Machine learning-based prediction and analysis of thermoelectric performance in cubic Na_3_Bi

To complement the insights obtained from density functional theory (DFT) and Boltzmann transport calculations, we employed machine learning (ML) regression models to predict the dimensionless thermoelectric figure of merit, *ZT*, from temperature-dependent transport properties of cubic Na_3_Bi. DFT gives us a microscopic view, while ML lets us quickly make predictions, analyse how sensitive features are to changes, and make predictions across a range of parameters. In this study, we utilised two supervised machine learning models: Random Forest and a fully connected Neural Network aiming to evaluate their predictive accuracy, interpretability, and conformity with established physical behaviour.

#### 3.5.1 Dataset construction and feature engineering

The dataset was created from first-principles calculations of the thermoelectric transport coefficients for cubic Na_3_Bi at three temperatures: 100 K, 500 K, and 950 K, which cover a wide range of temperatures, from very low to very high (Table 2). By taking samples from 101 chemical potentials between −1.5 eV and +1.5 eV, we got 101 data points for each temperature. We divided the dataset into two parts: 75% for training (76 points) and 25% for testing (25 points). We trained and tested models separately at each temperature. The input features for each temperature were:

Seebeck coefficient: *S* in μV K^−1^.

Electrical conductivity: *σ* in S m^−1^.

Thermal conductivity *κ* in W m^−1^ K^−1^.

**Table 2:** Estimated thermoelectric properties and dimensionless figure of merit (*ZT*) for tetragonal NaBi, hexagonal NaBi_3_, and cubic Na_3_Bi at 100 K, 500 K, and 950 K, using extracted Seebeck coefficient (S), electrical conductivity (*σ*), and electronic thermal conductivity (*κ*_e_). A constant lattice thermal conductivity of 1.5 W m^−1^ K^−1^ was assumed for all compounds

Compounds	Temperatures (*K*)	*S* (μV K^−1^)	*σ* (S m^−1^)	*κ*_e_ (W m^−1^ K^−1^)	*κ*_l_ (W m^−1^ K^−1^)	Total *κ* (W m^−1^ K^−1^)	*ZT*
Tetragonal NaBi	100	90	1.5 × 10^5^	5	1.5	6.5	0.19
	500	130	1.2 × 10^5^	16	1.5	17.5	0.29
	950	180	1.0 × 10^5^	22	1.5	23.5	0.13
Hexagonal NaBi_3_	100	40	8.0 × 10^4^	2.5	1.5	4.0	0.03
	500	70	6.0 × 10^4^	6	1.5	7.5	0.20
	950	90	5.0 × 10^4^	8	1.5	9.5	0.21
Cubic Na_3_Bi	100	650	1.0 × 10^5^	7	1.5	8.5	0.50
	500	400	1.1 × 10^5^	15	1.5	16.5	0.53
	950	500	1.0 × 10^5^	20	1.5	21.5	0.11

These features were selected because they influence thermoelectric efficiency and result from *ab initio* transport calculations. Even though *ZT* can be calculated analytically from these parameters, the ML models are made to learn how they interact with each other in a complex, nonlinear, and temperature-dependent way across chemical potential space. This lets us use SHAP values for efficient surrogate prediction and sensitivity analysis, providing both accuracy and interpretability that goes beyond the direct formula.

#### 3.5.2 Training strategy and model parameters

Each model was trained independently for each temperature using a 75%/25% train-test split. Input features for NN were standardized *via* z-score normalization.

Hyperparameters for both models were optimized *via* grid-based manual tuning. We changed the number of estimators for RF and used the mean squared error criterion to find the best balance between performance and complexity. We chose 100 trees as the best number. We used a two-layer dense architecture (16 neurons each) with ReLU activations and trained the NN with the Adam optimiser. To avoid overfitting, early stopping (patience = 20 epochs) was used. We chose these settings after testing RMSE and *R*^2^ on validation splits over and over again, which performed successfully given the dataset's size.

#### 3.5.3 Parity plots and predictive performance

**Fig. 10:**
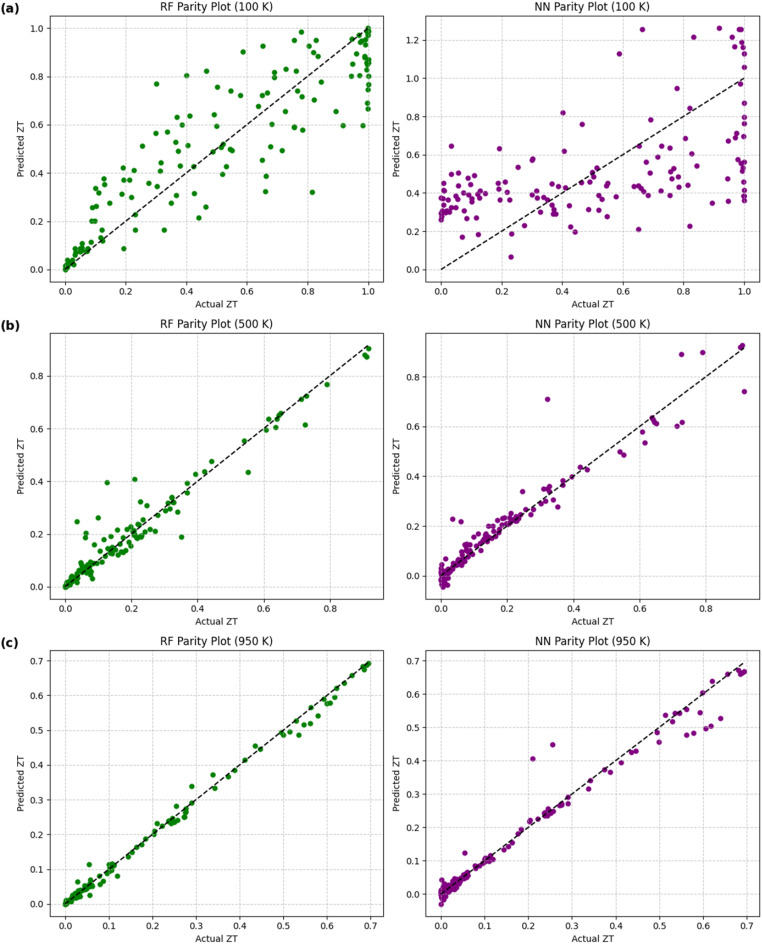
Parity plots showing predicted *versus* actual *ZT* values using Random Forest (left) and Neural Network (right) models at (a) 100 K, (b) 500 K, and (c) 950 K.

The performance of both models on the test set is summarized in Table 3. Parity plots (Fig. 10) also correspond to test predictions, showing the correlation between predicted and true *ZT* values. For clarity, we now include a comparison of training and test errors (*R*^2^ and RMSE). The fact that the training and test metrics are remarkably similar shows that both models work well in general, but RF is preferred due to its resistance to overfitting at low temperatures. The RF model consistently proved more accurate than the NN, especially at 100 K, where the NN did not fit the data well. The NN performed substantially better at higher temperatures (500 K and 950 K), almost matching the RF.

**Table 3:** Performance metrics (*R*^2^ and RMSE) of Random Forest and Neural Network models in predicting *ZT* for cubic Na_3_Bi at 100 K, 500 K, and 950 K using the test set. Models were trained on 75% of the data and evaluated on the remaining 25%

Temp (K)	Model	*R* ^2^ (train)	*R* ^2^ (test)	RMSE (train)	RMSE (test)
100	RF	0.984	0.971	0.122	0.160
100	NN	0.847	0.791	0.189	0.160
500	RF	0.995	0.994	0.037	0.049
500	NN	0.965	0.947	0.053	0.049
950	RF	0.998	0.998	0.012	0.014
950	NN	0.997	0.996	0.015	0.014

This trend shows that the transport properties become smoother and less noisy at higher temperatures. This is advantageous for models like NNs, which are sensitive to data noise. We observe that tree-based ensemble models, like RF, are ideal for small, structured datasets because they are resistant to overfitting. In contrast, the NN model performed better at higher temperatures, where the transport trends are smoother.

#### 3.5.4 SHAP analysis and feature sensitivity

We employed SHAP to interpret the RF model. SHAP values quantify the marginal contribution of each feature to the model output by computing Shapley values from cooperative game theory.

**Fig. 11:**
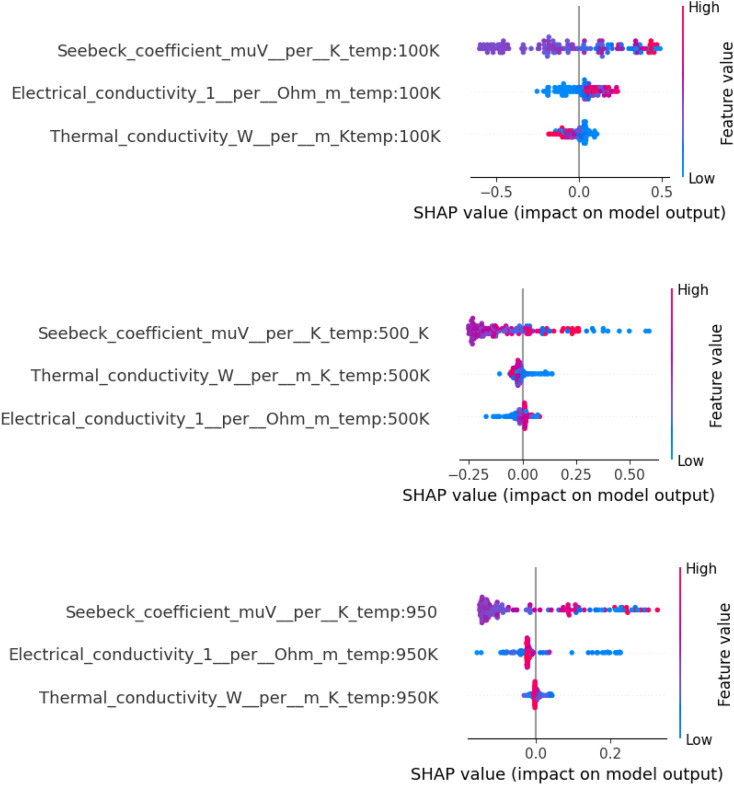
SHAP summary plots illustrating feature importance in predicting *ZT* at (top) 100 K, (middle) 500 K, and (bottom) 950 K.

Fig. 11 shows SHAP summary plots for each temperature. In all cases, the Seebeck coefficient was the most important factor, which is in line with its role as a quadratic term in the *ZT* formula. The SHAP values also showed the effect of *κ* at 100 K, since it changes more at low temperatures. At 950 K, σ was more important because it was the main factor in total heat transport and Joule heating losses at high temperatures.

These ML models not only give accurate *ZT* predictions, but they also give quantitative information about how to design materials. The SHAP and parity plots back up the well-known rules:

• At all temperatures, it is important to maximise the Seebeck coefficient.

• At high *T*, electrical conductivity becomes more important.

• Thermal conductivity, which is always inversely related to *ZT*, is more important at cryogenic temperatures where *κ* usually dominates the denominator.

• RF is better in this case because it can work with small structured datasets that do not have many features. NN performed similarly at high temperatures but tended to underfit at low temperatures. These findings indicate that RF is more appropriate for initial ML modelling of thermoelectrics, whereas neural networks may be advantageous for larger, noisier datasets with additional descriptors (*e.g.*, atomic structure, bonding metrics, *etc.*).

This machine learning framework can be readily extended to predict *ZT* for related materials (*e.g.*, Na_3_Bi alloys, strain-tuned structures, or doped variants). Incorporating additional features such as electronic density of states, effective masses, or band gap descriptors could further improve generalizability. Moreover, hybrid models combining tree-based learning with deep feature extraction (*e.g.*, graph neural networks) offer promising avenues for interpretable, data-driven thermoelectric design. This ability to understand, along with the ability of RF and NN models to make predictions, supports the idea that ML is not redundant but rather helpful, especially in complicated thermoelectric systems where the interactions between transport coefficients are not linear and depend on temperature.

The remarkable thermoelectric properties exhibited by Na_3_Bi in proximity to its Dirac point warrant a comparison with established theoretical models for Dirac materials. The Tang-Dresselhaus theory gives a full picture of how thermoelectric transport works in quantum-confined Dirac systems. It says that the Seebeck coefficients will be higher because of the unique linear dispersion and density of states near Dirac points.^66–70^ Our results in bulk 3D Na_3_Bi exhibit conceptually coherent behaviour; the exceptionally high Seebeck coefficients (reaching ±1200 μV/K) we observe arise from identical fundamental physics: linear band dispersion and the disappearance of density of states at the Dirac energy. While the material realization differs (3D bulk crystal *vs.* 2D quantum-confined systems), the underlying mechanism of enhanced thermoelectric response near Dirac points appears universal. Moreover, the integrated DFT-ML methodology developed in this work provides a general framework that can be directly applied to study thermoelectric properties in the Dirac materials described by the Tang-Dresselhaus theory. Our approach, which combines accurate electronic structure calculation with machine learning surrogate modeling, is particularly well-suited for exploring the complex parameter space of engineered Dirac systems, including doping, strain, and quantum confinement effects. The SHAP interpretability analysis further offers a data-driven means to validate theoretical predictions about feature importance in these systems.

## 4. Conclusion

This work provides a comprehensive multi-scale investigation of Na–Bi compounds by combining density functional theory and machine learning models. First-principles calculations elucidated the crystal structures, electronic topologies, phonon dynamics, and thermoelectric transport of tetragonal NaBi, hexagonal NaBi_3_, and cubic Na_3_Bi. Our results confirm that cubic Na_3_Bi exhibits superior thermoelectric potential at intermediate temperatures due to its enhanced Seebeck response and Dirac semimetallic nature. Additionally, by integrating machine learning models trained on DFT-calculated data, we demonstrated that Random Forest and Neural Network models can reliably predict *ZT* across varying temperatures, with RF models showing superior performance at low temperatures. The SHAP interpretability framework further clarified the relative feature importance, reinforcing known physical principles and providing data-driven insights. These findings showcase the power of ML in augmenting first-principles calculations, not only by predicting *ZT* efficiently, but also by quantifying feature contributions and offering a framework that is extensible to doped or unexplored Na–Bi derivatives. Future research may apply this methodology to Na_3_Bi alloys, doped systems, or strained structures, facilitating the interpretable and efficient design of topological thermoelectrics *via* hybrid physics-informed machine learning workflows. This framework can easily be used with other types of Dirac materials, such as the quantum-confined systems described by the Tang-Dresselhaus theory. This makes it possible to compare different Dirac material platforms.

## Conflicts of interest

There are no conflicts to declare.

## Data Availability

All data are available in the data repository ZENODO at: 10.5281/zenodo.15499220 (https://zenodo.org/records/15499220).
